# Ultrafast and Large-Scale Fabrication of PEDOT:PSS Nanofilms Using Electrical-Field-Assisted Direct Ink Deposition

**DOI:** 10.3390/molecules28165989

**Published:** 2023-08-10

**Authors:** Banashree Gogoi, Carson Gockley, Sushmitha Venu, Yizhen Zhu, Pranith Alluri, Ayinawu Abdul Malik, Mitesh Suhas Despande, Raveena Phadnis, Evangeline Amonoo, Xiangjia Li, Terry L. Alford

**Affiliations:** 1 School of Molecular Sciences, Arizona State University, Tempe, AZ 85287, USA; bgogoi2@asu.edu; 2 School for Electrical, Computer, and Energy Engineering, Arizona State University, Tempe, AZ 85287, USA; cpgockle@asu.edu; 3 School for Engineering of Matter, Transport and Energy, Arizona State University, Tempe, AZ 85287, USA; svenu2@asu.edu (S.V.); yzhu245@asu.edu (Y.Z.); palluri1@asu.edu (P.A.); aamalik4@asu.edu (A.A.M.); mdeshpa7@asu.edu (M.S.D.); eaamonoo@asu.edu (E.A.); 4 School of Computing and Augmented Intelligence, Arizona State University, Tempe, AZ 85287, USA; rphadnis@asu.edu

**Keywords:** PEDOT:PSS, conductive nanofilm, electrical field, direct ink deposition, spin coating

## Abstract

The importance of conductive polymers has significantly increased over the decade due to their various applications, such as in electronic devices, sensors, and photovoltaics. Poly(3,4-ethylene dioxythiophene) polystyrene sulfonate (PEDOT:PSS) is one of the most successfully and widely used polymers in practical applications. Spin coating is extensively used to fabricate these conductive films; however, it has disadvantages. It is inherently a batch process with relatively low output and high solution wastage. To address these issues, we developed a novel printing process called electrical-field-assisted direct ink deposition (EF-DID), which yields a continuous, homogenous film with high electrical conductivity. In this process, we studied the formation of nanodroplets under an electrical field and their effects on film characteristics. Furthermore, dimethyl sulfoxide (DMSO) was considered as an additive solvent to increase the conductivity and wettability of the films. We then compared EF-DID-printed PEDOT:PSS films with spin-coated films to better understand the film properties. Finally, inverted perovskite solar cell devices were fabricated and compared, where the PEDOT:PSS layers were prepared by EF-DID printing and spin coating. Based on the experimental results, a solution of 20% PEDOT:PSS in DMSO (vol/vol) printed by EF-DID for 15 s provided optimal morphology.

## 1. Introduction

Poly (3,4-ethylenedioxythiophene) polystyrene sulfonate (PEDOT:PSS) is considered one of the most successful conductive polymers, having great significance in commercial and fundamental research [1,2]. The excellent properties of PEDOT:PSS make it an exceptionally useful material, with wide applications, such as in transparent conductors, anti-static coatings, thermoelectric materials, etc. [3,4,5]. Compared to other conductive polymers, PEDOT:PSS has several advantages, such as high optical transparency, low material cost, high and tunable electrical conductivity, excellent thermal stability, and easy processing [6,7]. PEDOT:PSS has been employed extensively in light-emitting diodes, photovoltaics, and thin-film transistors as transparent electrodes [8,9,10,11]. Research shows that the different film processing, deposition, and post-processing steps impact the morphology and arrangement of the PEDOT:PSS nanostructures in the prepared thin films. Electronic and optical properties are also affected by the PEDOT:PSS nanostructures in the as-prepared thin films [12,13,14,15,16,17].

Various fabrication techniques, such as dip coating, spin coating, doctor blading, slot-die coating, ink-jet printing, gravure printing, screen printing, and spray coating, have been employed to deposit thin films and obtain different film characteristics [15,16,17]. The spin-coating method has been one of the most widely used techniques; however, it is inherently a non-scalable process that can only be used for fabrication in batches. Additionally, spin coating is characterized by high levels of material wastage of the liquid precursor. Thus, the large-scale production of thin films is not possible using spin coating. This paper reports a novel printing process for fabricating large-area nanofilms within minutes. We named the process electrical-field-assisted direct ink deposition (EF-DID). The EF-DID fabrication technique is a coating technique consisting of a grounded plate with a heated substrate targeted by electric-field-accelerated jets of liquid droplets transporting the reactants. The solution droplets move from the nozzle to the substrate. As the droplets leave the nozzle head, an electric charge is added. Inter-droplet repulsion causes them to deform into a conical shape called the Taylor cone, from the tip of which a fluid jet emerges. As the fluid jet travels and the solvent continuously evaporates, the droplet size decreases, and surface charge density increases. Electrostatic forces then override the surface tension, dispersing the droplets into a wide plume of smaller, more crystalline droplets. Finally, a solid thin film is formed when the solutes are deposited and solidify on the heated substrate. Integrating x-y-z movements and depositing patternable nanofilms with a material extrusion module combines direct ink printing into the electrical field production module. The conductive PEDOT:PSS thin films were quickly produced with EF-DID. The highest film quality could be achieved by optimizing printing parameters, such as applied voltage, solution concentration, deposition height, and ink flow rate. The voltage was maintained at about 12 kV to ensure smooth printing of thin films; higher voltage can lead to the instability of the Taylor cone.

Similarly, at low velocity, the proper formation of the cone can be ensured. Around 40 cm of distance is preserved for better film deposition. To achieve optimum film deposition, an ink flow rate of 2 µL/s is used, since a steady and evenly distributed deposit is essential for the fabrication of nanofilms. To achieve optimal film thickness, 20% PEDOT:PSS in DMSO (*v*/*v*) was printed for 15 s. Later, the films were annealed for 15 min at 130 °C to evaporate the remaining solvent and enhance the crystal growth.

The EF-DID-printed films were compared to the spin-coated PEDOT:PSS films in terms of morphology and distribution of the PEDOT:PSS nanostructures. We believe that the presented EF-DID technique will lower the production cost and be a highly efficient nanofilm printing technique for the fabrication of next-generation solar cells.

In this study, we intend to establish a reproducible and reliable technique for fabricating large-area thin films for perovskite solar cells. The EF-DID technique discussed in this manuscript will allow layer-by-layer film growth, resulting in a dense and uniform microstructure, compared to that of films fabricated by sputtering [18]. The reported deposition process could also be used to fabricate perovskite solar cells using 2D materials [19,20].

## 2. Results and Discussion

### 2.1. Film Morphology

**Figure 1:**
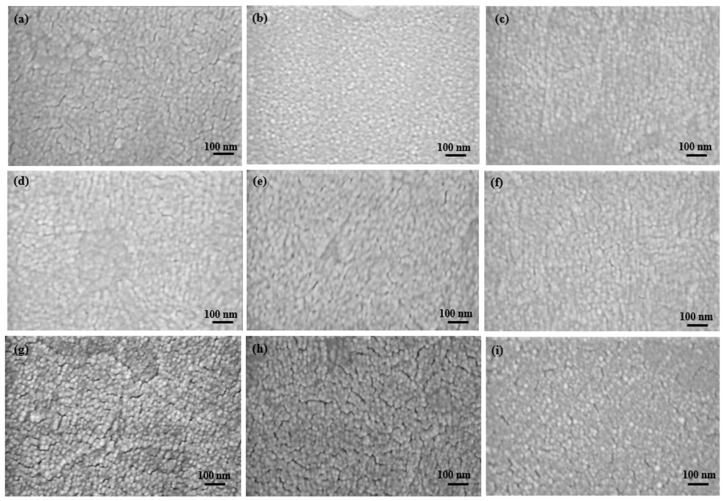
EF-DID-printed films of 10% PEDOT:PSS in DMSO (vol/vol): (**a**) 8 s; (**b**) 10 s; (**c**) 15 s. EF-DID-printed films of 20% PEDOT:PSS in DMSO (vol/vol): (**d**) 8 s; (**e**) 10 s; and (**f**) 15 s. EF-DID-printed films of 30% PEDOT:PSS in DMSO (vol/vol): (**g**) 8 s; (**h**) 10 s; and (**i**) 15 s.

The surface properties of the EF-DID-printed film were evaluated using scanning electron microscopy (SEM). The nanofilms were prepared on indium tin oxide (ITO) and annealed at 130 °C for 15 min. As can be seen in Figure 1, all the annealed PEDOT:PSS films had similar characteristics. Films printed with a solution of 10% PEDOT:PSS in DMSO (vol/vol) (Figure 1a–c), deposited for different times (8, 10, and 15 s, had an uneven surface coverage compared to the films with the other concentrations. The films printed with 20% PEDOT:PSS in DMSO (vol/vol) (Figure 1d–f) had the best film coverage with nanostructures. The 20% PEDOT:PSS in DMSO (vol/vol) (Figure 1f) film with a deposition time of 15 shad a very homogenous nanostructure with uniform coverage and equal grain sizes. Compared to the others, the films printed using 30% PEDOT:PSS in DMSO (vol/vol) (Figure 1g–i) were inhomogeneous and had many pinholes and cracks, even after annealing. Higher concentrations of PEDOT:PSS caused larger droplets and instability of the spray. This caused inhomogeneous film formation, leading to pinholes and cracks.

Thus, uniform crack-free homogenous films were achieved by adjusting the concentration of PEDOT:PSS ink. Our results showed that the film printed using 20% PEDOT:PSS in DMSO (vol/vol) for 15 swas the optimum film.

The as-deposited PEDOT:PSS films must be annealed every time after deposition. Annealing time and temperature are two critical aspects of PEDOT:PSS thin film formation. The annealing of the films is necessary to remove the excess solvent. In addition, annealing favors the formation of specific structural phases, improving the surface roughness of the films. Based on our previous studies, we employed 130 °C for 15 min in this manuscript [21]. Annealing the as-deposited EF-DID-fabricated films at this temperature and for this duration gave us an optimum thickness of around 60 nm.

Furthermore, the EF-DID-fabricated annealed films also showed an improved effective resistivity of 4.15 × 10^4^ ± 0.26 Ω-m. This study uses DMSO as an additional PEDOT:PSS solution solvent. Since DMSO has a high boiling point, it requires a higher annealing temperature for a longer time to evaporate the complete solvent from the thin films. Thus, annealing the EF-DID-fabricated PEDOT:PSS films at 130 °C for 15 min proved optimal for our films.

**Figure 2:**
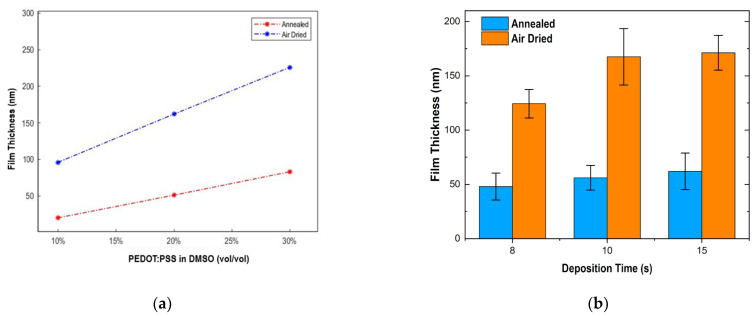
PEDOT:PSS film thickness: (**a**) vs. PEDOT:PSS in DMSO (vol/vol); and (**b**): vs. deposition time.

Furthermore, we investigated the EF-DID process for large-scale production of PEDOT:PSS nanofilm in our previous work [21]. The thicknesses of the deposited PEDOT:PSS nanofilm were evaluated using a stylus profilometer. The nanofilm thicknesses were assessed as a function of the concentration of PEDOT:PSS in DMSO (vol/vol) and the deposition time (Figure 2a,b). It can be seen that nanofilm thickness increased with increasing PEDOT:PSS concentration in DMSO (vol/vol). As seen in Figure 2b, the nanofilms were EFA-DID printed using different deposition times. Film thickness increased with deposition time. EFA-DID printing for 15 sgave the thickest films, at 60 nm. Both annealed (130 °C) and air-dried films were tested to identify the optimal postprocessing. The nanofilm thickness increased with increasing PEDOT:PSS concentration in DMSO (vol/vol), as well as with deposition time. Moreover, we also observed a decrease in thickness with the annealing of the nanofilm, presumably due to water loss and the intraparticle interactions between the molecules at higher temperatures. The deposition rate can also be seen to decrease with overall deposition time in Figure 2b. This can be attributed to a surface charge buildup on the substrate that is inherent to the EF-DID process. Liquid droplets that impact the substrate rather than the grounding electrode do not have a conductive path for neutralizing charge accumulation. The resultant electrostatic field repels some incoming droplets, effectively lowering the deposition rate.

### 2.2. Film Wettability

An effective resistivity of 4.15 × 10^4^ ± 0.26 Ω-m was calculated for all of the different concentrations of annealed films (10%, 20%, 30% PEDOT:PSS in DMSO (vol/vol)) [18].

**Figure 3:**
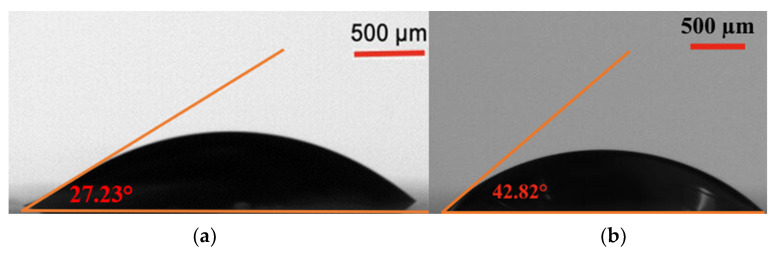
Contact angle of nanofilm: microscope photograph of water droplet on (**a**) EF-DID-printed PEDOT:PSS nanofilm and (**b**) spin-coated PEDOT:PSS nanofilm.

**Figure 4:**
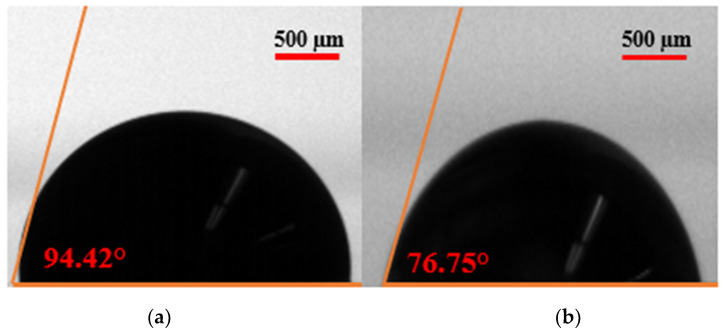
Contact angle of nanofilm: microscope photograph of (**a**) PEDOT:PSS on ITO and (**b**) PEDOT:PSS in DMSO on ITO.

EF-DID-printed PEDOT:PSS films have been used as the hole transport layer (HTL) in perovskite solar cells. The hydrophobicity of the HTL determined the morphology of the active perovskite layer [22]. Consequently, the wettability of the EF-DID-printed film was calculated. A water droplet was added to the films, and the contact angle was determined. Figure 3a shows that the EF-DID-printed PEDOT:PSS nanofilm had a contact angle of 27.23°. This seems to be an ideal angle for spraying the Perovskite layer to prepare solar cells. By comparison, the spin-coated film (shown in Figure 3b) had a higher contact angle of 42.82°. These results indicate that the wettability of EF-DID-printed PEDOT:PSS films is improved compared to samples prepared by spin coating. Furthermore, this difference may be attributable to the difference in film surface roughness between the deposition techniques. The hydrophobic nature of PEDOT:PSS is believed to be decreased when using DMSO, a hydrophilic solvent [23]. Figure 4 supports this claim, showing that the contact angle of PEDOT:PSS on ITO decreases when DMSO is present. Thus, the EF-DID-printed PEDOT:PSS hydrophobicity can be lowered by including DMSO in the nano-ink used during film deposition.

### 2.3. EF-DID-Sprayed vs. Spin-Coated Nanofilms

**Figure 5:**
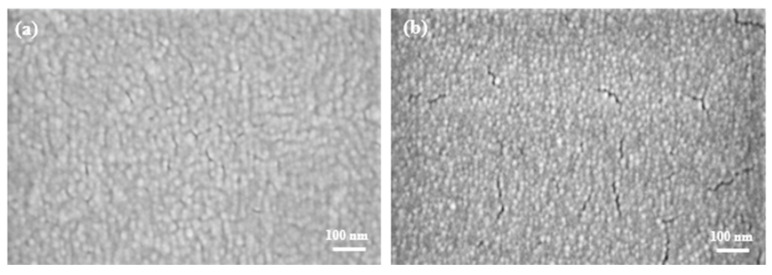
PEDOT:PSS nanofilm: (**a**) EF-DID sprayed; (**b**) spin coated.

This work aims to achieve a high-quality PEDOT:PSS film for future optoelectronic use. Since spin coating is one of the most commonly used fabrication methods, the following section compares EF-DID-sprayed and spin-coated nanofilms. SEM was performed on spin-coated and EF-DID-printed PEDOT:PSS nanofilms (Figure 5). As seen in Figure 5a, the EF-DID-printed nanofilm (20% PEDOT:PSS in DMSO (vol/vol) for 15 s) was a more compact film than the spin-coated film. Additionally, much fewer pinholes and cracks were observed for the EF-DID-printed nanofilm; this suggested a more homogenous film formation.

**Figure 6:**
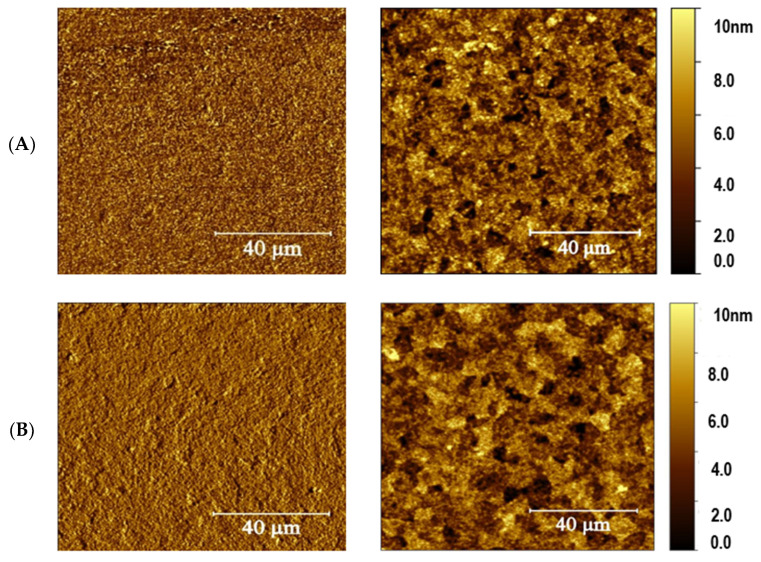
AFM phase and height images of (**A**) EF-DID-printed and (**B**) spin-coated PEDOT:PSS.

Further atomic force microscopy (AFM) analysis was performed on both the spin-coated and EF-DID-printed films. Figure 6 shows the differences in surface profile and topography for both the EF-DID-printed and spin-coated PEDOT:PSS nanofilms. According to one study, the spinning speed in the spin-coating method has a smoothening effect on the film’s morphology and structure [24]. The RMS was 2.03 nm for the spin-coated and 1.91 nm for the EF-DID-printed PEDOT:PSS nanofilms. Although spin coating had the advantage with respect to its smoothing effect, the surface roughness of our EF-DID-printed nanofilm (Figure 6a) was almost the same as that of the spin-coated film. However, spin coating had a disadvantage compared to EF-DID-printed nanofilms: the centrifugal forces applied to the PEDOT:PSS solution during spinning caused phase separation. As a result, the PEDOT layer formed the film’s bottom layer, and the PSS layer formed the film’s surface [24].

**Figure 7:**
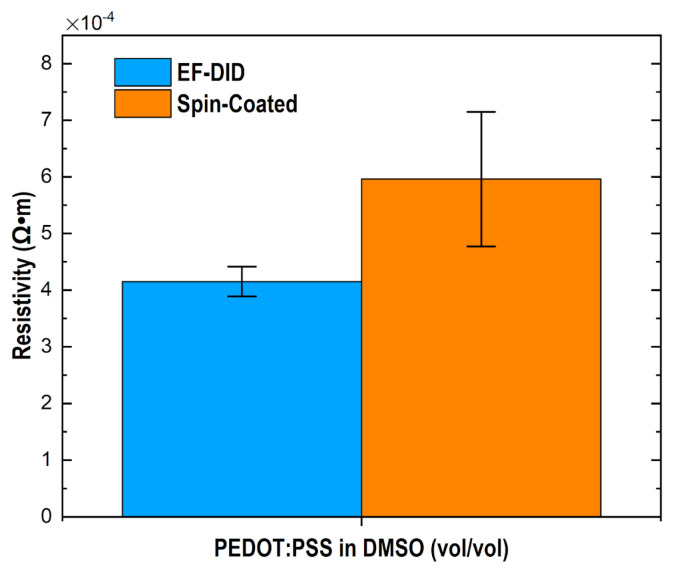
Resistivity of spin-coated and EF-DID-coated PEDOT:PSS in DMSO (vol/vol).

As indicated in Figure 6b, a self-assembled PSS-rich layer results in a smooth spin-coated PEDOT:PSS film [25]. The PSS chain of PEDOT:PSS is acidic and contains a sulfonic group causing degradation of the film’s morphology and stability [26,27]. The slight difference in RMS roughness between the spin-coated and EF-DID-printed nanofilms is the result of the different solvents used in preparing the PEDOT:PSS solution. In our case, the EF-DID-printed nanofilms contained 20% PEDOT:PSS in DMSO (vol/vol). The roughness increased slightly with increasing concentrations of DMSO due to the formation of tiny grains of PEDOT:PSS [27]. An effective resistivity of 4.15 × 10^4^ ± 0.26 Ω-m was calculated for all of the different concentrations of annealed films (10%, 20%, 30% PEDOT:PSS in DMSO (vol/vol)) [21]. An Ossila four-point probe was used to measure the sheet resistance of the EF-DID-printed nanofilms. Compared to EF-DID-coated PEDOT:PSS films, the spin-coated layer had a higher effective resistivity of 5.96 × 10^4^ ± 0.20 Ω-m (Figure 7). This can be attributed to the highly dense nature of the EF-DID-printed film compared to the spin-coated film.

**Figure 8:**
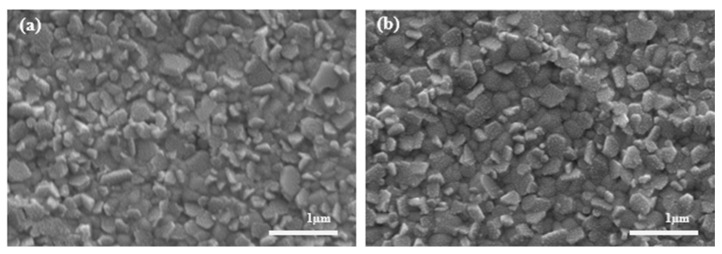
CH_3_NH_3_PbI_3_ spin coated on top of (**a**) EF-DID-printed PEDOT:PSS and (**b**) spin-coated PEDOT:PSS.

Both EF-DID-printed and spin-coated PEDOT:PSS nanofilms were evaluated as HTLs in perovskite solar cells. To test the bonding between the HTL and the perovskite layer, we further spin coated 1 M CH_3_NH_3_PbI_3_ (in a molar ratio of CH_3_NH_3_I:PbI_2_ = 1:0.8) on top of the EF-DID-printed and spin-coated PEDOT:PSS nanofilms (Figure 8). SEM characterization was performed to compare the morphology of the perovskite films. As shown in Figure 8, the perovskite film on top of the EF-DID-printed PEDOT:PSS layer looked more homogenous and compact than the other.

The current–voltage characteristics of the prepared inverted PSC devices were measured. Prior to the measurement, the light source was calibrated with a standard Si photodiode reference cell. By analyzing the J-V data, various parameters such as short-circuit current (Isc), open-circuit voltage (Voc), fill factor (FF), and efficiency can be determined. These parameters can provide valuable insights into the device’s performance and help assess its suitability for solar energy conversion applications.

**Figure 9:**
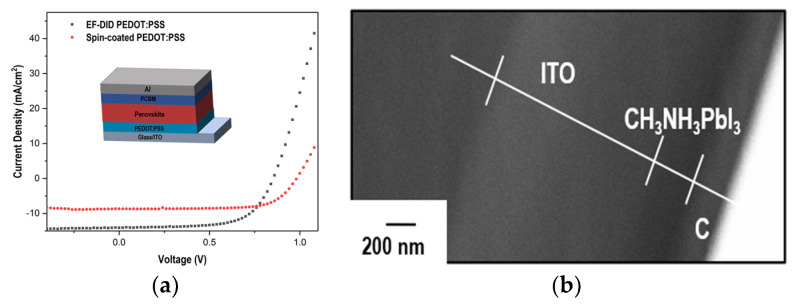
(**a**) J-V characteristics of inverted PSC with the spin-coated and EF-DID printed PEDOT:PSS layers; (**b**) cross-section image of spin-coated CH_3_NH_3_PbI_3_ (C: Au coating).

Figure 9a and Table 1 show the data for the inverted PSC devices fabricated using the spin-coating and EF-DID techniques. The PSC produced by spin coating exhibited a PCE of 6.0% with a Voc of 0.98 V, a Jsc of 8.7 mA/cm^2^, and an FF of 70%. The PSCs fabricated using the EF-DID technique significantly improved the PCE by ~30% to 7.8% (Voc = 0.85 V, Jsc = 14 mA/cm^2^, and FF = 65%). The spin-coated CH_3_NH_3_PbI_3_ film has a thickness measuring around 200 nm, as seen in the cross-section image in Figure 9b.

The shunt resistance (Rsh) and series resistance (Rs) are two important parameters for explaining the device characteristics of solar cells. Rs represents the resistance of the current flow within the series circuit of the solar cell. It includes the resistance of the conductive materials, such as metal contacts and interconnects, and the resistance of the semiconductor material itself. Rs should be as low as possible in order to minimize power losses within the solar cell. The Rsh represents the resistance path parallel to the solar cell’s main current flow, which indicates the relative amount of leakage current. In an ideal case, Rsh should be as high as possible. Inspection of Table 1 shows that the EF-DID-coated sample has a better Rs; however, it has a higher amount of leakage current. This may be associated with the porosity of the prepared sample.

As shown in Figure 9a, a large reverse/leakage current can be observed for the spin-coated devices. In Figure 5b, the spin-coated PEDOT:PSS sample has more pin holes and cracks compared to the EF-DID-fabricated PEDOT:PSS. These defects result in recombination and, subsequently, a large leakage current. The modification of the fabrication and spin-coating parameters is one of the ways to avoid this leakage of current.

## 3. Materials and Methods

### 3.1. Electrical-Field-Assisted Direct Ink Deposition of PEDOT:PSS

**Figure 10:**
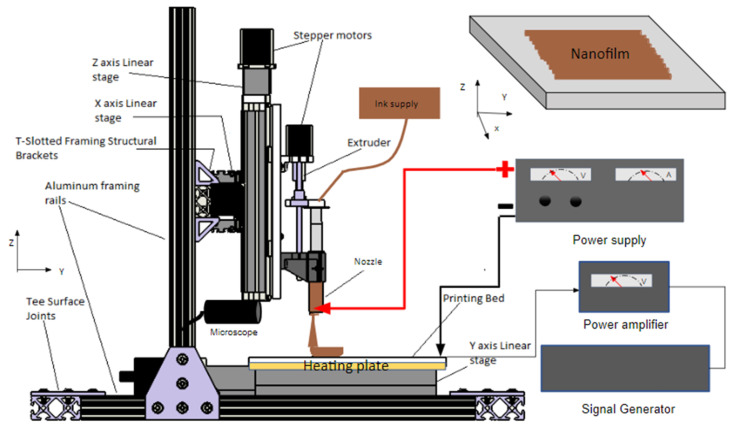
Schematic diagram of the EF-DID process for nanofilm fabrication [21].

The nanoscale printing technique, EF-DID, uses a prototype printer, which includes a heated x-y-z printing stage, a control module, an electrical field generator, and a material feed (Figure 10). The EF-DID approach consists of a material extrusion module, where the nano ink is fed into the plunger, pushed by the linear actuator. The extrusion flow rate of the nano ink is controlled by adjusting the speed of the linear actuator. A high-voltage power supply (0 to 30 kV) connected to the nozzle and grounded printing stage produces the electric field around the printing nozzle, and thus the Taylor cone necessary for EF-DID. Adjusting the distance and the voltage between the printing stage and the nozzle changes the electric field strength. However, care must be taken to ensure that the Taylor cone and ink plume remain stable during printing. In order to do so, a microscope was mounted to the printer to monitor the morphology of the printed liquid drops and the Taylor cone formed under the nozzle. This setup enables the large-scale fabrication of nanofilms with 2D patterns.

We studied the relation between the electrical field and the deposition parameters (e.g., the speed setting) to understand the EF-DID technique’s printing diameter. A semi-spherical shape was developed at the tip of the nozzle when applying a high voltage V, and an electrical field was induced [28]:

(1) $$E=\frac{2V}{\alpha \ln\left(\frac{4H}{\alpha }\right)},$$

where E is the electrical field strength, α is the liquid radius of the curvature, and H is the distance between the nozzle and the printing substrate. As the voltage is raised, the liquid deforms from the semi-spherical shape into a conical shape with a fluid jet at the tip. This is recognized as the Taylor cone. Due to the charges of the droplets, they repel each other in the fluid jet and form a plume. The strength of the repulsive force and travel distance to the substrate then determine the printing diameter.

The print size was evaluated according to the deposition diameter D, and was adjusted by controlling the distance between the printing substrate and the nozzle. The diameter was directly proportional to the increase in distance, and inversely proportional to the deposition thickness. Therefore, the relationship between the diameter and the distance between the printing substrate and the nozzle was as follows:

(2) D=τH,

where τ is a constant 0.1794 for an applied voltage of 10 kV. The equation below determines the thickness of deposition, T_d_:

(3) $$T_{d\;}=\frac{4\gamma t}{{\pi D}^{2}},$$

where γ is the flow rate and t is the time.

### 3.2. Ink Preparation

PEDOT:PSS dispersed in water was purchased from Ossila Al 4083, Sheffield, UK. DMSO was used to dilute the ink and was purchased from Sigma Aldrich, Darmstadt, Germany. PEDOT:PSS in DMSO (vol/vol) solutions with different concentrations (10%, 20%, and 30%) were prepared. The diluted solutions were then sonicated for about 10 min for better dispersion. Finally, the solution prepared as described above was filtered using a 0.45 µm filter to remove undispersed nanoparticles before printing.

### 3.3. Thin Film Fabrication

**Figure 11:**
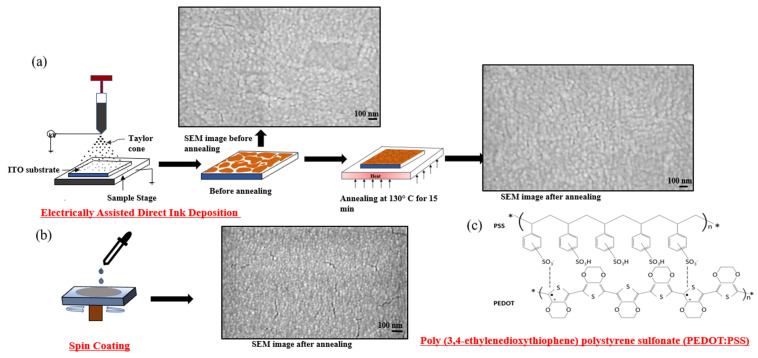
Schematic diagrams of PEDOT:PSS nanofilm fabrication: (**a**) EF-DID; (**b**) spin coating; and (**c**) chemical structures of PEDOT:PSS used in this work.

Initially, ITO substrates were sequentially cleaned using an ultrasonic treatment sequence of deionized water, acetone, and IPA, for 15 min each. Previous work has shown that polar solvents with high dielectric constants and boiling points improve the electrical conductivity of PEDOT:PSS [29]. Here, we used DMSO to further dilute the PEDOT:PSS solution. Three solutions of PEDOT:PSS in DMSO (vol/vol)—10%, 20%, and 30%—were printed to evaluate the performance of the nanofilms. The diluted PEDOT:PSS ink was loaded into a plastic syringe with a stainless-steel needle with a blunt tip (Bstean 32 G 13 mm, 0.09 mm inner diameter, and 0.25 mm outer diameter). The substrates were placed onto an aluminum printing stage equipped with a linear actuator with a 150 mm range (purchased from the Parker-Hannifin Corporation, Mayfield Heights, OH, USA). A silicon heating pad with a thermistor (NTC 100 K thermistor) was attached to the printing stage to heat the substrates during and after printing. All the EF-DID setup operations were controlled using a Duet 2 control board. The RepRap Firmware configuration tool was used to configure the control system for setup. An HV350REG high-powered voltage generator was used to produce an electrical field to initiate film deposition. Based on experimental considerations, the flow rate was set at 0.64 µL/s and the voltage was set at 10 kV. Different deposition times were used. After printing, the PEDOT:PSS films were annealed at 130 °C for 15 min (Figure 11a). The morphologies of the as-prepared films were studied to find the best film with the most-improved performance.

For spin coating, which is a common technique applied for fabricating thin films using centripetal force and the surface tension of the solution, the PEDOT:PSS solution was mixed with 5% DMSO and spin coated onto an ITO substrate at 4000 rpm for 60 s(Figure 11b). Then, the film was annealed at 130 °C for 15 min.

### 3.4. Characterization

Both the EF-DID-printed and spin-coated samples were characterized using various techniques to understand the properties of the thin films. The thicknesses of the prepared nanofilms were evaluated using a stylus profilometer (DEKTAK XT, Bruker, Billerica, MA, USA). An SEM XL-30 Environmental FEG scanning electron microscope (JEOL, Peabody, MA, USA) was used to study the film morphology. The root-mean-square (RMS) roughness of the films was characterized using an SPM Bruker Multimode (MM8, Billerica, MA, USA) atomic force microscope (AFM). An Ossila four-point probe was used to measure the sheet resistance. To test the hydrophobicity of the prepared films, contact angle measurements were performed using a Minder Hightech SDC-350, Guangzhou, China. Current–voltage measurements (J-V) of the prepared PVS devices were performed under simulated AM 1.5 G (100 mW/cm^2^) radiation using a Xenon lamp (Spectra-Physics, Oriel Instruments, Milpitas, CA, USA).

## 4. Conclusions

A homogenous PEDOT:PSS nanofilm was achieved using a high-speed printing process named electrical-field-assisted direct ink deposition. DMSO solvent was used with PEDOT:PSS to improve the conductivity of the nanofilms. Experiments were conducted to relate the parameters of EF-DID to the nanofilm properties. As a result, the desired thickness and the best morphology of the PEDOT:PSS nanofilms were achieved by printing 20% PEDOT:PSS in DMSO (vol/vol) for 15 s. Moreover, a comparison between the spin-coated and EF-DID-printed nanofilms was performed, where the EF-DID-printed nanofilm outperformed the spin-coated one in terms of homogeneity and morphology. Overall, the EF-DID process shows prospects for the highly efficient fabrication of nanofilms.

The EF-DID fabrication technique offers the opportunity for large-scale fabrication of perovskite solar cells under ambient conditions. The more commonly used spin-coating fabrication techniques face significant challenges in terms of scaling them up for large-volume fabrication. Broader research must focus on other strategies that enable stable open-air perovskite solar cell fabrication. The proposed fabrication technique facilitates the fabrication of different photovoltaic materials for the hole transport layer (HTL), electron transport layer (ETL), and active perovskite layer in the open air. Furthermore, it could increase the scientific community’s interest in making further advances in research and development for the 3D printing of nanofilms. In addition, flexible electronics could also be developed using the EF-DID technique for further research on novel energy devices.

## Figures and Tables

**Table 1:** Device parameters of inverted PSC devices with the spin-coated and EF-DID-printed PEDOT:PSS layer.

Deposition Type	Efficiency (%)	Fill Factor	V_oc_ (V)	J_sc_ (mA/cm^2^)	R_sh_ (Ωcm^2^)	R_s_ (Ωcm^2^)
Spin Coated PEDOT:PSS	6.0	0.709	0.983	8.67	2857	15
EF-DID PEDOT:PSS	7.8	0.647	0.853	14.2	1003	5.5

## Data Availability

Not applicable.

## References

[1] Groenendaal L., Jonas F., Freitag D., Pielartzik H., Reynolds J.R. (2000). Poly(3,4-Ethylenedioxythiophene) and Its Derivatives: Past, Present, and Future. Adv. Mater..

[2] Yue R., Xu J. (2012). Poly(3,4-Ethylenedioxythiophene) as Promising Organic Thermoelectric Materials: A Mini-Review. Synth. Met..

[3] Elschner A., Kirchmeyer S., Lovenich W., Merker U., Reuter K. (2010). PEDOT: Principles and Applications of an Intrinsically Conductive Polymer.

[4] Perepichka I.F., Perepichka D.F. (2009). Handbook of Thiophene-Based Materials: Applications in Organic Electronics and Photonics.

[5] Zhang B., Sun J., Katz H.E., Fang F., Opila R.L. (2010). Promising Thermoelectric Properties of Commercial PEDOT:PSS Materials and Their Bi _2_ Te _3_ Powder Composites. ACS Appl. Mater. Interfaces.

[6] Yano H., Kudo K., Marumo K., Okuzaki H. (2019). Fully Soluble Self-Doped Poly(3,4-Ethylenedioxythiophene) with an Electrical Conductivity Greater than 1000 S cm^−1^. Sci. Adv..

[7] Ouyang J. (2013). “Secondary Doping” Methods to Significantly Enhance the Conductivity of PEDOT:PSS for Its Application as Transparent Electrode of Optoelectronic Devices. Displays.

[8] Patra A., Bendikov M., Chand S. (2014). Poly(3,4-Ethylenedioxyselenophene) and Its Derivatives: Novel Organic Electronic Materials. Acc. Chem. Res..

[9] Greczynski G., Kugler T., Salaneck W.R. (1999). Characterization of the PEDOT-PSS System by Means of X-Ray and Ultraviolet Photoelectron Spectroscopy. Thin Solid Films.

[10] Yildiz S., Cai J.L., Fan Q.G. (2012). Effects of Solvents on the Electrical Resistance of Poly(3,4-Ethylenedioxythiophene) on Textiles. Adv. Mater. Res..

[11] Kim Y.H., Sachse C., Machala M.L., May C., Müller-Meskamp L., Leo K. (2011). Highly Conductive PEDOT:PSS Electrode with Optimized Solvent and Thermal Post-Treatment for ITO-Free Organic Solar Cells. Adv. Funct. Mater..

[12] Kirchmeyer S., Reuter K. (2005). Scientific Importance, Properties and Growing Applications of Poly(3,4-Ethylenedioxythiophene). J. Mater. Chem..

[13] Kemerink M., Timpanaro S., De Kok M.M., Meulenkamp E.A., Touwslager F.J. (2004). Three-Dimensional Inhomogeneities in PEDOT:PSS Films. J. Phys. Chem. B.

[14] Xia Y., Ouyang J. (2011). PEDOT:PSS Films with Significantly Enhanced Conductivities Induced by Preferential Solvation with Cosolvents and Their Application in Polymer Photovoltaic Cells. J. Mater. Chem..

[15] Eslamian M., Newton J. (2014). Spray-on PEDOT:PSS and P3HT:PCBM Thin Films for Polymer Solar Cells. Coatings.

[16] Krebs F.C. (2009). Fabrication and Processing of Polymer Solar Cells: A Review of Printing and Coating Techniques. Sol. Energy Mater. Sol. Cells.

[17] Cho C.-K., Hwang W.-J., Eun K., Choa S.-H., Na S.-I., Kim H.-K. (2011). Mechanical Flexibility of Transparent PEDOT:PSS Electrodes Prepared by Gravure Printing for Flexible Organic Solar Cells. Sol. Energy Mater. Sol. Cells.

[18] Yu X., Smith J., Zhou N., Zeng L., Guo P., Xia Y., Alvarez A., Aghion S., Lin H., Yu J. (2015). Spray-combustion synthesis: Efficient solution route to high-performance oxide transistors. Proc. Natl. Acad. Sci. USA.

[19] Dastgeer G., Afzal A.M., Nazir G., Sarwar N. (2021). p-GeSe/n-ReS2 Heterojunction Rectifier Exhibiting a Fast Photoresponse with Ultra-High Frequency-Switching Applications. Adv. Mater. Interfaces.

[20] Dastgeer G., Nisar S., Shahzad Z.F., Rasheed A., Kim D., Jaffery S.H.A., Wang L., Usman M., Eom J. (2023). Low-Power Negative-Differential-Resistance Device for Sensing the Selective Protein via Supporter Molecule Engineering. Adv. Sci..

[21] Zhu Y., Gogoi B., Alluri P., Suhas Despande M., Hutchins J., Tagbor E., Alford T.L., Li X. (2022). 3D Printing of Largescale Functional Nanofilm Using Electrically Assisted Direct Ink Deposition. Manuf. Lett..

[22] Zhou H., Chen Q., Li G., Luo S., Song T., Duan H.-S., Hong Z., You J., Liu Y., Yang Y. (2014). Interface Engineering of Highly Efficient Perovskite Solar Cells. Science.

[23] Lee S.H., Sohn J.S., Kulkarni S.B., Patil U.M., Jun S.C., Kim J.H. (2014). Modified Physico–Chemical Properties and Supercapacitive Performance via DMSO Inducement to PEDOT:PSS Active Layer. Org. Electron..

[24] Zabihi F., Xie Y., Gao S., Eslamian M. (2015). Morphology, Conductivity, and Wetting Characteristics of PEDOT:PSS Thin Films Deposited by Spin and Spray Coating. Appl. Surf. Sci..

[25] Ramizy A., Hassan Z., Omar K., Al-Douri Y., Mahdi M.A. (2011). New Optical Features to Enhance Solar Cell Performance Based on Porous Silicon Surfaces. Appl. Surf. Sci..

[26] Cameron J., Skabara P.J. (2020). The Damaging Effects of the Acidity in PEDOT:PSS on Semiconductor Device Performance and Solutions Based on Non-Acidic Alternatives. Mater. Horiz..

[27] Reza K.M., Mabrouk S., Qiao Q. (2018). A Review on Tailoring PEDOT:PSS Layer for Improved Performance of Perovskite Solar Cells. Proc. Nat. Res. Soc..

[28] Loeb L.B., Kip A.F., Hudson G.G., Bennett W.H. (1941). Pulses in Negative Point-to-Plane Corona. Phys. Rev..

[29] Kim J.Y., Jung J.H., Lee D.E., Joo J. (2002). Enhancement of Electrical Conductivity of Poly(3,4-Ethylenedioxythiophene)/Poly(4-Styrenesulfonate) by a Change of Solvents. Synth. Met..

